# Prolonged pre-firing pancreatic compression with linear staplers in distal pancreatectomy: a valuable technique for post-operative pancreatic fistula prevention

**DOI:** 10.1007/s00423-024-03350-8

**Published:** 2024-06-12

**Authors:** Giuseppe Quero, Vito Laterza, Carlo Alberto Schena, Giuseppe Massimiani, Chiara Lucinato, Claudio Fiorillo, Teresa Mezza, Flavia Taglioni, Roberta Menghi, Ludovica Di Cesare, Beatrice Biffoni, Davide De Sio, Fausto Rosa, Vincenzo Tondolo, Sergio Alfieri

**Affiliations:** 1 Gemelli Pancreatic Center, CRMPG (Advanced Pancreatic Research Center), Fondazione Policlinico Universitario “Agostino Gemelli” IRCCS, Largo Agostino Gemelli 8, Rome, 00168 Italy; 2 Università Cattolica del Sacro Cuore di Roma, Largo Francesco Vito 1, Rome, 00168 Italy; 3 Pancreas Unit, Medicina Interna e Gastroenterologia, CEMAD Centro Malattie dell’Apparato Digerente, Fondazione Policlinico Universitario “Agostino Gemelli” IRCCS, Largo Agostino Gemelli 8, Rome, 00168 Italy; 4 General Surgery Unit, Fatebenefratelli Isola Tiberina – Gemelli Isola, Via di Ponte Quattro Capi, 39, Rome, 00186 Italy; 5 Pancreatic Surgery Unit, Department of Surgery, Fondazione Policlinico Universitario “Agostino Gemelli”, IRCCS, Largo Agostino Gemelli, 8, Rome, 00168 Italy

**Keywords:** Distal pancreatectomy, Post-operative pancreatic fistula, Pre-firing compression, Stapler compression

## Abstract

**Purpose:**

Post-operative pancreatic fistula (POPF) remains the main complication after distal pancreatectomy (DP). The aim of this study is to evaluate the potential benefit of different durations of progressive stapler closure on POPF rate and severity after DP.

**Methods:**

Patients who underwent DP between 2016 and 2023 were retrospectively enrolled and divided into two groups according to the duration of the stapler closure: those who underwent a progressive compression for < 10 min and those for ≥ 10 min.

**Results:**

Among 155 DPs, 83 (53.5%) patients underwent pre-firing compression for < 10 min and 72 (46.5%) for ≥ 10 min. As a whole, 101 (65.1%) developed POPF. A lower incidence rate was found in case of ≥ 10 min compression (34–47.2%) compared to < 10 min compression (67- 80.7%) (*p* = 0.001). When only clinically relevant (CR) POPFs were considered, a prolonged pre-firing compression led to a lower rate (15–20.8%) than the < 10 min cohort (32–38.6%; *p* = 0.02). At the multivariate analysis, a compression time of at least 10 min was confirmed as a protective factor for both POPF (OR: 5.47, 95% CI: 2.16–13.87; *p* = 0.04) and CR-POPF (OR: 2.5, 95% CI: 1.19–5.45; *p* = 0.04) development. In case of a thick pancreatic gland, a prolonged pancreatic compression for at least 10 min was significantly associated to a lower rate of CR-POPF compared to < 10 min (*p* = 0.04).

**Conclusion:**

A prolonged pre-firing pancreatic compression for at least 10 min seems to significantly reduce the risk of CR-POPF development. Moreover, significant advantages are documented in case of a thick pancreatic gland.

## Introduction

Distal pancreatectomy (DP) currently represents the gold standard of treatment for benign and malignant lesions of the body and tail of the pancreas. Although DP is widely recognized as an easier procedure to perform in comparison with pancreaticoduodenectomy, surgery-related morbidity remains high [1, 2]. Post-operative pancreatic fistula (POPF) represents the most concerning adverse event with an incidence rate up to 60% and a clinically relevant (CR-POPF) variant reported in up to 35% of DPs [3–6]. POPF occurrence has been associated with the potential onset of additional complications, such as hemorrhage, intra-abdominal abscesses, and death in most severe cases [7, 8]. While several patient-related factors have been recognized a predisposing to POPF development (i.e. obesity, young age, non-malignant lesions, a thick and soft pancreatic parenchyma) [9–14], scarce and contrasting data are present on the optimal technique of pancreatic transection during DP. Some authors [15] did not highlight any statistically significant difference in terms of POPF rate between the stapler closure and hand-sewn technique, while others reported the stapler closure as superior to other techniques for preventing POPF [16, 17]. For instance, the traumatic transection of pancreatic ductules is one of the recognized risk features for POPF development [18, 19] and, theoretically, the mechanical stapling technique should be able to seal those pancreatic ductules lowering POPF incidence rate. On the counterpart, staplers are rigid and sharp devices, and their use may cause a mechanical trauma of the pancreatic gland and ductules leading to a potentially higher rate of POPF. In this controversial context, only few studies evaluated the potential benefits deriving from a progressive pre-firing closure of the stapler [20–22]. The hypothesis is that the progressive compression of the pancreatic parenchyma should be able to reduce the gland injury, aiding at the same time the sealing of pancreatic ductules.

Based on this postulation, we here present our retrospective experience on the application of a progressive stapler closure in DP with two different durations of the pre-firing pancreatic compression, with the main aim of evaluating the impact of such technique on the incidence rate of POPF.

## Materials and methods

After Institution Review Board (IRB) approval, all patients who underwent a DP at the Pancreatic Surgery Unit of the Fondazione Policlinico Universitario “Agostino Gemelli” IRCCS of Rome between January 2016 and July 2023 were retrospectively enrolled in the study. Only DPs performed with the use of a triple-layer endo stapler were included in the analysis. The application of fibrin glue or absorbable fibrin collagen sealant sponge on the transection margin were considered exclusion criteria. Clinico-demographic characteristics collected included: age, sex, body mass index (BMI), American Society of Anesthesiologists (ASA) score, diabetes, neoadjuvant therapy and pathological diagnosis. The intraoperative features analyzed were: the type of surgical approach (open, laparoscopic or robot-assisted), operative time and estimated blood loss (EBL), transection site (pancreatic body or isthmus), type of procedure (with or without splenectomy), pancreatic texture (soft or hard), Wirsung diameter (≤ or > 3 mm), pancreas thickness and stapler closure duration. Pancreatic texture was defined as hard or soft intraoperatively by the operating surgeon, while Wirsung diameter and pancreatic thickness were retrieved from the histopathological reports. Post-operative complications were registered and classified according to the Clavien-Dindo grading system [23], while POPF was defined and graded according to the 2016 International Study Group of Pancreatic Surgery (ISGPS) classification [24]. Length of hospital stay (LOS) and 30-day mortality were also reported.

For the study purposes, patients were categorized into two groups according to the median pre-firing stapler closure duration of the entire cohort: those who underwent a progressive pre-firing compression for less than 10 min constituted the control group (< 10 min group), while those with a compression duration of 10 min or longer constituted the study group (≥ 10 min group).

### Surgical procedure

All DPs were performed by two senior surgeons (S.A. and R.M.). An open, laparoscopic or robot-assisted approach was proposed by the operating surgeon according to the lesion type, location and expected surgical complexity. In all cases, the pancreatic gland was transected with a triple-layer stapler. Only the Echelon Flex™(Ethicon Endosurgery, Cincinnati, OH) with a black cartridge (staple size 4.4 mm) was used. Stapler closure duration varied according to the operating surgeon’s preference, ranging from 2 min (the minimum value reported in the literature to have benefits in terms of POPF rate [22]) to 15 min. Specifically, the pancreas was progressively compressed directly with Echelon and then the stapler was fired. An additional 2 min of post-firing compression was then applied.

Drainage tubes placed during surgery were used for POPF monitoring, and removed when the amylase content became lower than three times the upper normal limit of serum amylase, or when the output was almost null.

### Study outcomes

The main endpoint of the study was to compare the two cohorts of patients categorized according to the stapler closure duration (< 10 min or ≥ 10 min) in terms of POPF rate and severity. Moreover, a multivariate analysis of the potential influencing factors on POPF (including biochemical leak (BL)) and CR-POPF onset was additionally performed. Furthermore, a comparative analysis between the two groups was performed according to the pancreatic thickness, in order to evaluate the potential benefits of the progressive stapler compression on CR-POPF occurrence.

### Statistical analysis

All continuous data were reported as median and quartile rank (QR) while numbers and percentages were used for all categorical data. Univariate analysis included Mann-Whitney U test, Student’s t-tests, χ2 test, and Fisher’s exact test.

Univariate and multivariate logistic regression analyses were undertaken to identify risk factors for POPF (including biochemical leak (BL)) and CR-POPF (only grade B and C) development. Risk estimates were approximated as odds ratios (ORs) with 95% confidence intervals (CI). A receiver operating characteristics (ROC) analysis was used to analyze the efficacy of pancreatic thickness in POPF prediction and determine its cut-off value. For this purpose, patients were divided in those with a CR-POPF (grade B/C) and those without CR-POPF (no-POPF/BL group). An area under the curve (AUC) greater than 0.8 was considered of high diagnostic accuracy. The cut-off level was identified at an optimized accuracy with equal weight given to the errors of sensitivity and specificity [25]. The cut-off value was used for CR-POPF rate comparison according to the stapling times. For all tests, a *p*-value ≤ 0.05 was considered statistically significant. All data were analyzed using SPSS for Windows, version 25 (SPSS Inc., Chicago, IL, United States).

## Results

During the study period, 169 patients underwent DP. Of them, 14 (8.3%) were excluded from the analysis: 4 patients due to transection of the pancreas using a scalpel and subsequent hand-sewn closure of the pancreatic remnant, and 10 due to the application of fibrin glue (6 patients) or sealant sponge (4 patients) on the pancreatic resection margin after parenchyma transection with a linear stapler. Therefore, a total of 155 patients were retrospectively enrolled in the study. The majority of patients underwent DP for pancreatic ductal adenocarcinoma (PDAC). Intraoperatively, a soft pancreatic texture and a Wirsung diameter ≤3 mm were encountered in 89 (57.4%) and 84 (54.2%) cases, respectively, while median pancreatic thickness was 23 [19–26] mm. The median pre-firing stapler closure time was 10 [5-13.75] minutes. Accordingly, 72 (46.5%) patients had a pre-firing pancreatic compression for less than 10 min while 83 (53.5%) for 10 min or more. Post-operatively, POPF occurred in 101 (65.1%) patients. Specifically, 54 (32.2%) developed a BL, while a CR-POPF (grade B and C) was reported in 47 (28.4%) patients (Table 1).

**Table 1:** Clinico-demographic and perioperative features

***Clinico-demographic characteristics***	
**Age, years median (QR)**	64 (55–73)
**Sex, n (%)**	
*Male*	73 (47.1)
*Female*	82 (52.9)
**BMI, kg/m** ^**2**^ **median (QR)**	24.3 (22.5–28)
**ASA score, n (%)**	
*I*	19 (12.3)
*II*	101 (65.2)
*III*	35 (22.6)
**Diabetes, n (%)**	29 (18.7)
**Neoadjuvant therapy, n (%)**	10 (6.5)
**Pathological diagnosis, n (%)**	
*PDAC*	82 (52.9)
*IPMN*	25 (16.1)
*Neuroendocrine tumor*	37 (23.9)
*Mucinous cystadenoma*	11 (7.1)
***Intraoperative features***	
**Surgical approach, n (%)**	
*Open*	63 (40.6)
*Laparoscopic*	36 (23.2)
*Robot-assisted*	56 (36.1)
**Site of pancreatic transection, n (%)**	
*Body*	121 (78.1)
*Isthmus*	34 (21.9)
**Type of surgical procedure**	
*With splenectomy*	141 (91)
*Spleen-preserving*	14 (9)
**Operative time, min median (QR)**	240 (195–280)
**EBL, mL median (QR)**	235 (115–413)
**Vascular resection, n (%)**	15 (9.7)
**Pancreas texture, n (%)**	
*Hard*	66 (42.6)
*Soft*	89 (57.4)
**Wirsung diameter, n (%)**	
*≤3 mm*	84 (54.2)
*> 3 mm*	71 (45.8)
**Pancreas thickness, mm median (QR)**	23 (19–26)
**Stapling time, min median (QR)**	10 (5-13.75)
**Stapling time groups, min n (%)**	
*≥ 10*	83 (53.5)
*< 10*	72 (46.5)
***Postoperative course***	
**Clavien-Dindo complications ≥3, n (%)**	50 (32.2)
**POPF, n (%)**	101 (65.1)
**POPF grade n (%)**	
*BL*	54 (34.8)
*B*	44 (28.4)
*C*	3 (1.9)
**LOS, days median (QR)**	7 (6–9)
**30-day mortality, n (%)**	2 (1.3)

BMI: body mass index; ASA: American Society of Anesthesiologists; PDAC: pancreatic ductal adenocarcinoma; IPMN: Intraductal Papillary Mucinous Neoplasm; EBL: estimated blood loss; POPF: post-operative pancreatic fistula; LOS: length of hospital stay

### POPF rate and clinical course according to the stapler closure time (Table 2)

**Table 2:** Clinico-demographic characteristics and postoperative course according to the stapling time

	Stapling time			
	< 10 min (*n*:83)	≥ 10 min (*n*:72)	*p*	
***Clinico-demographic characteristics***				
**Age, years median (QR)**	65 (57–74)	62 (54–72)	0.21	
**Sex, n (%)**				
*Male*	41 (49.4)	32 (44.4)	0.53	
*Female*	42 (50.6)	40 (55.6)		
**BMI, kg/m** ^**2**^ **median (QR)**	24.3 (22.7–28.1)	24.4 (22.4–27.5)	0.8	
**ASA score, n (%)**				
*I*	9 (10.8)	10 (13.9)	0.41	
*II*	58 (69.9)	43 (59.7)		
*III*	16 (19.3)	19 (26.4)		
**Diabetes, n (%)**	15 (18.1)	14 (19.4)	0.82	
**Neoadjuvant therapy, n (%)**	5 (6)	5 (6.9)	0.81	
**Pathological diagnosis, n (%)**				
*PDAC*	52 (62.7)	30 (41.7)	0.05	
*IPMN*	11 (13.3)	14 (19.4)		
*Neuroendocrine tumor*	14 (16.9)	23 (31.9)		
*Mucinous cystadenoma*	6 (7.2)	5 (6.9)		
***Intraoperative features***				
**Surgical approach, n (%)**				
*Open*	36 (43.4)	27 (37.5)	0.44	
*Laparoscopic*	16 (19.3)	20 (27.8)		
*Robot-assisted*	31 (37.3)	25 (34.7)		
**Site of pancreatic transection, n (%)**				
*Body*	65 (78.3)	56 (77.8)	0.93	
*Isthmus*	18 (21.7)	16 (22.2)		
**Type of surgical procedure**				
*With splenectomy*	78 (94)	63 (87.5)	0.16	
*Spleen-preserving*	5 (6)	9 (12.5)		
**Operative time, min median (QR)**	240 (185–285)	250 (205–280)	0.17	
**EBL, mL median (QR)**	231 (112–314)	245 (190–413)	0.18	
**Vascular resection, n (%)**	9 (10.8)	6 (8.3)	0.59	
**Pancreas texture, n (%)**				
*Hard*	40 (48.2)	26 (36.1)	0.13	
*Soft*	43 (51.8)	46 (63.9)		
**Wirsung diameter, n (%)**				
*≤3 mm*	46 (55.4)	38 (52.8)	0.74	
*> 3 mm*	37 (44.6)	34 (47.2)		
**Pancreas thickness, mm median (QR)**	24 (2–27)	22 (17–25)	0.2	
***Postoperative course***				
**Clavien-Dindo complications≥3, n (%)**	24 (28.9)	26 (36.1)	0.2	
**POPF (including BL), n (%)**	67 (80.7)	34 (47.2)	0.001	
**POPF grade, n (%)**				
*BL*	35 (42.2)	19 (26.4)	0.008	
*B*	30 (36.1)	14 (19.4)		
*C*	2 (2.4)	1 (1.4)		
**CR-POPF, n (%)**	32 (38.6)	15 (20.8)	0.02	
**LOS, days median (QR)**	7 (6–9)	7 (6–9)	0.52	
**30-day mortality, n (%)**	2 (2.4)	0	0.18	

BMI: body mass index; ASA: American Society of Anesthesiologists; PDAC: pancreatic ductal adenocarcinoma; IPMN: Intraductal Papillary Mucinous Neoplasm; EBL: estimated blood loss; POPF: post-operative pancreatic fistula; CR-POPF: clinically relevant post-operative pancreatic fistula; LOS: length of hospital stay

Demographic features were comparable between the two groups, except for a higher rate of pancreatic ductal adenocarcinoma (PDAC) in the < 10 min group (52–62.7% vs. 30–41.7% in the ≥ 10 min cohort; *p* = 0.05). No difference was noted in terms of indication to surgery, type of surgical approach and procedure, and operative time. Similarly, pancreatic texture, Wirsung diameter and pancreatic thickness were comparable between the two populations.

An inverse proportion was evidenced between the stapler closure duration and POPF occurrence, with a lower rate for a pre-firing closure duration of 10 min or longer. Specifically, a stapler closure duration < 10 min led to a significantly higher rate of POPF (67 patients – 80.7%) as compared to the ≥ 10 min cohort (34 patients – 47.2%). When the comparative analysis was performed according to POPF severity, the < 10 min group presented a significantly higher rate of both BL (35–42.2% vs. 19 -26.4%; *p* = 0.008) and CR-POPF (32–38.6% vs. 15–20.8%; *p* = 0.02).

### Risk factor analysis for POPF (including BL) and CR-POPF

At the univariate analysis (Table 3), none of the clinico-demographic characteristics was recognized as a risk feature for POPF (including BL). Furthermore, no difference was recorded in terms of type of pancreatic disease, surgical approach, site of pancreatic transection and spleen preservation between patients with and without POPF. Conversely, a soft pancreatic texture was a risk factor when compared to a firm pancreas (68–67.3% vs. 33–32.7%; *p* = 0.001). Similarly, a Wirsung diameter ≤3 mm (*p* = 0.005) and a pancreatic thickness of ≥ 23 mm (*p* < 0.0001) were related to higher incidence of POPF. Regarding the pre-firing stapling duration, a progressive closure ≥ 10 min was associated to a significantly lower rate of POPF (34–33.7%) as compared to a compression of < 10 min (67–66.3%) (*p* = 0.001).

**Table 3:** Prognostic factors analysis for POPF (including BL) in the entire study cohort

	POPF- (*n*:54)	POPF + (*n*:101)	*p*	Multivariate analysis (Logistic regression) odds ratio [95% Confidence Interval]	Multivariate *p* value
***Clinico-demographic characteristics***					
**Age, years median (QR)**	67 (60–74)	63 (55–72)	0.22		
**Sex, n (%)**					
*Male*	22 (40.7)	51 (50.5)	0.24		
*Female*	32 (59.3)	50 (49.5)			
**BMI, kg/m** ^**2**^ **median (QR)**	24 (22.1–28)	24.8 (22.9–27.8)	0.27		
**ASA score, n (%)**					
*I*	5 (9.2)	14 (13.9)	0.69		
*II*	36 (66.7)	65 (64.4)			
*III*	13 (24.1)	22 (21.8)			
**Diabetes, n (%)**	9 (16.7)	20 (19.8)	0.63		
**Neoadjuvant therapy, n (%)**	5 (9.3)	5 (5)	0.29		
**Pathological diagnosis, n (%)**					
*PDAC*	27 (50)	55 (54.5)	0.59		
*Non-PDAC*	27 (50)	46 (45.5)			
***Intraoperative features***					
**Surgical approach, n (%)**					
*Open*	23 (42.6)	40 (39.6)	0.59		
*Laparoscopic*	10 (18.5)	26 (25.7)			
*Robot-assisted*	21 (38.9)	35 (34.7)			
**Site of pancreatic transection, n (%)**					
*Body*	42 (77.8)	79 (78.2)	0.95		
*Isthmus*	12 (22.2)	22 (21.8)			
**Type of surgical procedure**					
*With splenectomy*	46 (85.2)	79 (78.2)	0.07		
*Spleen-preserving*	8 (14.8)	22 (21.8)			
**Operative time, min median (QR)**	231 (190–274)	250 (195–290)	0.29		
**EBL, mL median (QR)**	229 (125–310)	244 (195–210)	0.27		
**Vascular resection, n (%)**	6 (11.1)	9 (8.9)	0.65		
**Pancreas texture, n (%)**					
*Hard*	33 (61.1)	33 (32.7)	0.001	3.24 [1.18–8.86]	0.02
*Soft*	21 (38.9)	68 (67.3)			
**Wirsung diameter, n (%)**					
*≤3 mm*	21 (38.9)	63 (62.4)	0.005	0.98 [0.37–2.56]	0.97
*> 3 mm*	33 (61.1)	38 (37.6)			
**Pancreas thickness**^**a**^, **mm, n (%)**					
*≥ 23*	9 (16.7)	73 (72.3)	< 0.0001	12.6 [4.94–32.1]	< 0.0001
*< 23*	45 (83.3)	28 (27.7)			
**Stapling time**^**a**^, **min n (%)**					
*< 10*	16 (29.6)	67 (66.3)	0.001	5.47 [2.16–13.87]	0.04
*≥ 10*	38 (70.4)	34 (33.7)			

^a^The median value of pancreas thickness and stapling time were used as cut off for the univariate and multivariate analyses

POPF: post-operative pancreatic fistula; BMI: body mass index; ASA: American Society of Anesthesiologists; PDAC: pancreatic ductal adenocarcinoma; EBL: estimated blood loss

When included in the multivariate model, only a soft pancreatic texture (OR: 3.24, 95% CI:1.18–8.86; *p* = 0.02), a pancreatic thickness ≥ 23 mm (OR: 12.6, 95% CI: 4.94–32.1; *p* < 0.0001), and a prolonged stapling duration (OR: 5.47, 95% CI: 2.16–13.87; *p* = 0.04) were recognized as independent prognostic factors for POPF development (Table 3).

An additional analysis on the influencing factors for only CR-POPF (grade B and C) was also performed (Table 4). At the univariate analysis, patients with a soft pancreatic texture presented a higher incidence of CR-POPF (33 patients – 70.2%) in comparison to a firm gland (14 cases – 29.8%) (*p* = 0.03). Similarly, a thick pancreatic parenchyma was associated to a higher rate of CR-POPF (33 patients – 70.2%) as compared to a prancreatic thickness < 23 mm (14 patients – 51.1%) (*p* = 0.004). Furthermore, a progressive stapler closure for at least 10 min lead to a lower rate of CR-POPF, with 15 cases (31.9%) vs. 32 cases (68.1%) in the < 10 min group (*p* = 0.02).

**Table 4:** Prognostic factors analysis for CR-POPF in the entire study cohort

	CR-POPF – (*n*:108)	CR-POPF + (*n*:47)	*p*	Multivariate analysis (Logistic regression) odds ratio [95% Confidence Interval]	Multivariate *p* value
***Clinico-demographic characteristics***					
**Age, years median (QR)**	65 (56-73.5)	63 (55–72)	0.48		
**Sex, n (%)**					
*Male*	50 (46.3)	23 (48.9)	0.76		
*Female*	58 (53.7)	24 (51.1)			
**BMI, kg/m** ^**2**^ **median (QR)**	24.3 (22.5–27.8)	24.8 (22.5–29)	0.9		
**ASA score, n (%)**					
*I*	12 (11.1)	7 (14.9)	0.41		
*II*	74 (68.5)	27 (57.4)			
*III*	22 (20.4)	13 (27.7)			
**Diabetes, n (%)**	18 (16.7)	11 (23.4)	0.32		
**Neoadjuvant therapy, n (%)**	7 (6.5)	3 (6.4)	0.98		
**Pathological diagnosis, n (%)**					
*PDAC*	53 (49.1)	29 (61.7)	0.14		
*Non-PDAC*	55 (50.9)	18 (38.3)			
***Intraoperative features***					
**Surgical approach, n (%)**					
*Open*	44 (40.7)	19 (41.4)	0.63		
*Laparoscopic*	23 (21.3)	13 (27.7)			
*Robot-assisted*	41 (38)	15 (31.9)			
**Site of pancreatic transection, n (%)**					
*Body*	86 (79.6)	35 (74.5)	0.47		
*Isthmus*	22 (20.4)	12 (25.5)			
**Type of surgical procedure**					
*With splenectomy*	99 (91.7)	42 (89.4)	0.64		
*Spleen-preserving*	9 (8.3)	5 (10.6)			
**Operative time, min median (QR)**	240 (190–279)	250 (200–287)	0.48		
**EBL, mL median (QR)**	219 (136–310)	241 (195–215)	0.31		
**Vascular resection, n (%)**	9 (8.3)	6 (12.8)	0.39		
**Pancreas texture, n (%)**					
*Hard*	52 (48.1)	14 (29.8)	0.03	2.03 [1.19–4.5]	0.02
*Soft*	56 (51.9)	33 (70.2)			
**Wirsung diameter, n (%)**					
*≤3 mm*	56 (51.9)	28 (59.6)	0.37		
*> 3 mm*	52 (48.1)	19 (40.4)			
**Pancreas thickness**^**a**^, **mm, n (%)**					
*≥ 23*	49 (45.4)	33 (70.2)	0.004	2.26 [1.04–4.9]	0.01
*< 23*	59 (54.6)	14 (51.1)			
**Stapling time**^**a**^, **min n (%)**					
*< 10*	51 (47.2)	32 (68.1)	0.02	2.5 [1.19–5.45]	0.04
*≥ 10*	57 (52.8)	15 (31.9)			

^a^The median value of pancreas thickness and stapling time were used as cut off for the univariate and multivariate analyses

CR-POPF: clinically relevant post-operative pancreatic fistula; BMI: body mass index; ASA: American Society of Anesthesiologists; PDAC: pancreatic ductal adenocarcinoma; EBL: estimated blood loss

At the multivariate analysis for CR-POPF, a soft pancreatic texture (OR: 2.03; 95% CI: 1.19–4.5; *p* = 0.02), a thick pancreas (OR:2.26; 95% CI: 1.04–4.9; *p* = 0.01), and a prolonged pre-firing compression (OR: 2.5; 95% CI: 1.19–5.45; *p* = 0.04) were independent prognostic factors for a more severe manifestation of POPF.

### Optimal pre-firing pancreatic compression time according to pancreatic thickness

**Fig. 1:**
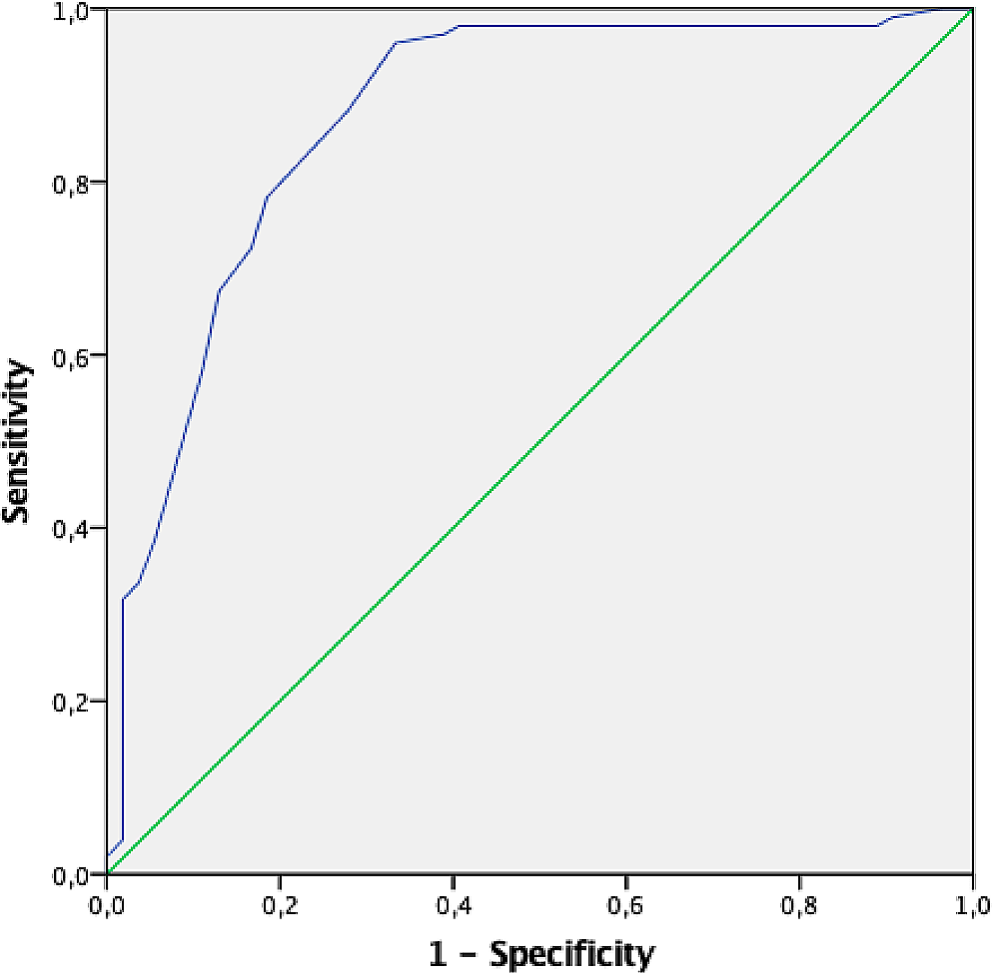
Receiver operating characteristics (ROC) curve for pancreas thickness (area under the curve (AUC): 0.87; 95% CI: 0.82–0.91). Clinically relevant post-operative pancreatic fistula (grade B/C fistulas) was the outcome variable

**Fig. 2:**
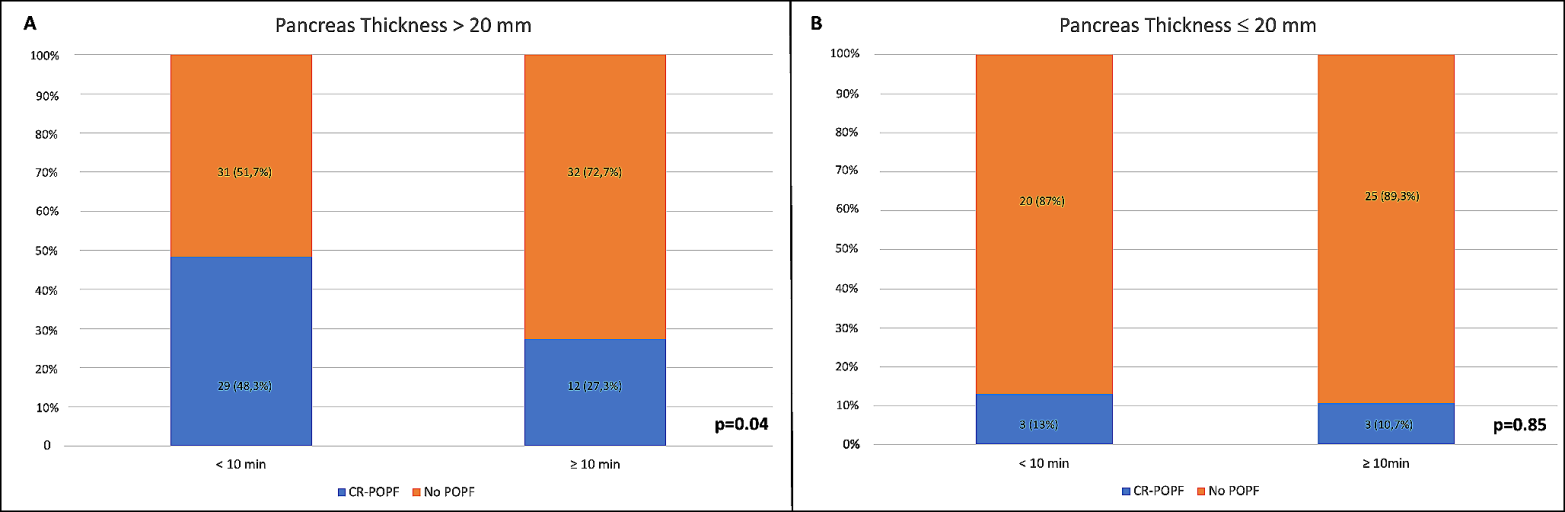
Optimal stapler closure time according to the cut-off for pancreatic thickness. (**A**) Comparative analysis for pancreatic thickness > 20 mm; (**B**) Comparative analysis for pancreatic thickness ≤20 mm

To identify a cut-off thickness value for the risk of CR-POPF occurrence, a ROC curve analysis was conducted (Fig. 1) and a value higher than 20 mm was identified as strongly associated to a higher risk (area under the curve: 0.87; sensitivity: 88%, specificity:72%). A comparative analysis was, thus, performed between the two groups of stapler closure time according to the above-mentioned cut-off for CR-POPF onset. As reported in Fig. 2A, a prolonged pancreatic compression led to a significantly lower incidence rate of CR-POPF, with the best outcomes in case of a compression of 10 min or longer (*p* = 0.04) Conversely, in case of pancreatic thickness ≤ 20 mm, the incidence rate of CR-POPF was not significantly dissimilar between the two groups of pre-firing compression time (Fig. 2B).

## Discussion

The use of linear staplers is the most frequent method for pancreatic transection in DP. However, the potential benefits of this methodology over other techniques is still highly debated. Although several authors focused their research on the optimal type of stapler and best cartridge to employ [26–30], only few studies, with no conclusive evidences, are currently present on the potential role that a progressive pre-firing compression may have on POPF onset [20–22]. Our retrospective analysis demonstrated a significantly lower rate of POPF with the increasing closure time of the linear stapler. In particular, a prolonged pre-firing compression led to a significant lowering of CR-POPF rate in case of a thick pancreatic parenchyma.

POPF is known as the most frequent post-DP adverse event, with an incidence rate that did not vary over the decades. Among the pancreas-related factors, a soft pancreatic texture and a thick pancreatic gland have been identified as non-modifiable risk characteristics [10–12]. These same factors have been confirmed as predisposing to POPF in our case series. Specifically, a soft pancreatic texture was confirmed as an independent risk feature for both POPF (OR: 3.24; 95%CI: 1.18–8.86; *p* = 0.02) and CR-POPF (OR: 2.03; 95%CI: 1.19–4.5; *p* = 0.02). Similarly, pancreatic thickness was a strong predictor of POPF in our series (AUC: 0.87), with a cut off level selected at 20 mm (sensitivity: 88%, specificity: 72%; OR: 13.9; 95% CI: 5.64–30.13). In this case, a thicker pancreatic gland was recognized as independent risk factor for POPF (OR: 12.6; 95% CI: 4.94–32.1; *p* < 0.0001) and CR-POPF (OR: 2.26; 95% CI: 1.04–4.9; *p* = 0.01).

Given the key role of these pancreas-related no-modifiable risk factors, it is of paramount importance to identify specific treatment strategies aimed to lower POPF incidence rate. Theoretically, the progressive pre-firing stapler closure should guarantee a lower trauma on pancreatic parenchyma, permitting at the same time a gradual sealing of pancreatic ductules. In this regard, Nakamura et al. and Ariyarathenam et al. [21, 22] demonstrated a significant advantage in terms of POPF occurrence with a progressive stapler closure of 3 min. Similarly, Okano et al. [20] routinely used a slowly pancreas compression for more than 5 min before firing, reporting a clinical advantage in terms of POPF rate.

Nevertheless, several limitations of these studies need to be addressed. First, the majority of them is based on limited case series. Secondly, no deep insight is given on the optimal pre-firing compression duration in relation to pancreas characteristics. In our case series, we provided an outcome analysis for two different compression durations. Interestingly, the incidence rate of POPF decreased with the prolongation of the stapler closure duration, with an occurrence rate reduced nearly by a half in case of a pre-firing closure of at least 10 min. When stratified for POPF severity, a prolonged stapler closure significantly influenced the incidence rate of BL (*p* = 0.008) and CR-POPF (*p* = 0.02). In order to avoid potential biases deriving from concomitant risk factors of POPF, we additionally performed a multivariate analysis, and a prolonged stapler closure was confirmed as an independent protective factor for both POPF and CR-POPF onset, with a 5.47-fold and 2.5-fold reduced risk, respectively.

To date, no author specifically evaluated the role of the progressive stapler closure in relation to pancreatic thickness. To accomplish this purpose, after deriving the cut-off value of pancreatic thickness for CR-POPF, we evaluated the hypothetical protective role of a progressive stapler closure on the base of the pancreas thickness. Interestingly, in case of a thick gland (more than 2 cm), the progressive stapler closure for at least 10 min led to a lower incidence rate of CR-POPF as compared to < 10 min (*p* = 0.04). Conversely, the stapler closure duration did not seem to have an impact on the onset of CR-POPF (*p* = 0.85) in case of pancreatic thickness inferior to 2 cm. These data would strongly suggest the clinical benefits deriving from a prolonged stapler closure, especially in presence of a thick pancreatic gland. In this last case, a progressive compression for at least 10 min would be highly suggested.

Despite this is the largest study present in the literature on the potential correlation among specific stapler closure timings and POPF development, several limitations need to be underlined. First, the retrospective study design and the consequent potential selection bias may affect the generalization of the results. Secondly, there was no specific indication for the duration of stapler closure to be employed. Moreover, the additional subdivision of patients according to pancreatic thickness, together with the categorization by duration, significantly reduced the sample sizes of analysis making difficult to draw definitive conclusions. In addition, the use a black cartridge independently of the pancreas characteristics may represent a further bias. Indeed, previous evidences [30–32] demonstrated major benefits in terms of POPF rate when the type of cartridge employed was chosen according to the pancreas thickness. Thus, this may have potentially influenced the POPF rate independently of the prefiring compression duration applied.

## Conclusion

In conclusion, a prolonged pre-firing pancreatic compression for at least 10 min seems to have a positive impact on the reduction of POPF (including BL) and CR-POPF rates after DP. In addition, major benefits have been shown especially in case of a thick pancreatic gland, reducing the incidence of CR-POPFs. Nevertheless, it is necessary to design prospective randomized trials in order to further corroborate our results.

## Data Availability

No datasets were generated or analysed during the current study.
